# Supplementary material for: Plasmodium translocon component EXP2 facilitates hepatocyte invasion
Source: Nat Commun. 2020 Nov 6;11:5654. doi: 10.1038/s41467-020-19492-4 (PMC7648069; doi:10.1038/s41467-020-19492-4)
Supplement: Supplementary file 1 — Supplementary Information [file 41467_2020_19492_MOESM1_ESM.pdf]

## Supplemental Information

for

# *Plasmodium* translocon component EXP2 facilitates hepatocyte invasion

João Mello-Vieira, Francisco J. Enguita, Tania F. de Koning-Ward, Vanessa Zuzarte-Luís*, Maria M. Mota*

*Correspondence to: vluis@fm.ul.pt; mmota@fm.ul.pt

File contains:

**Supplementary Figures and Legends**

**Statistical Test Table**

# Supplementary Figure 1

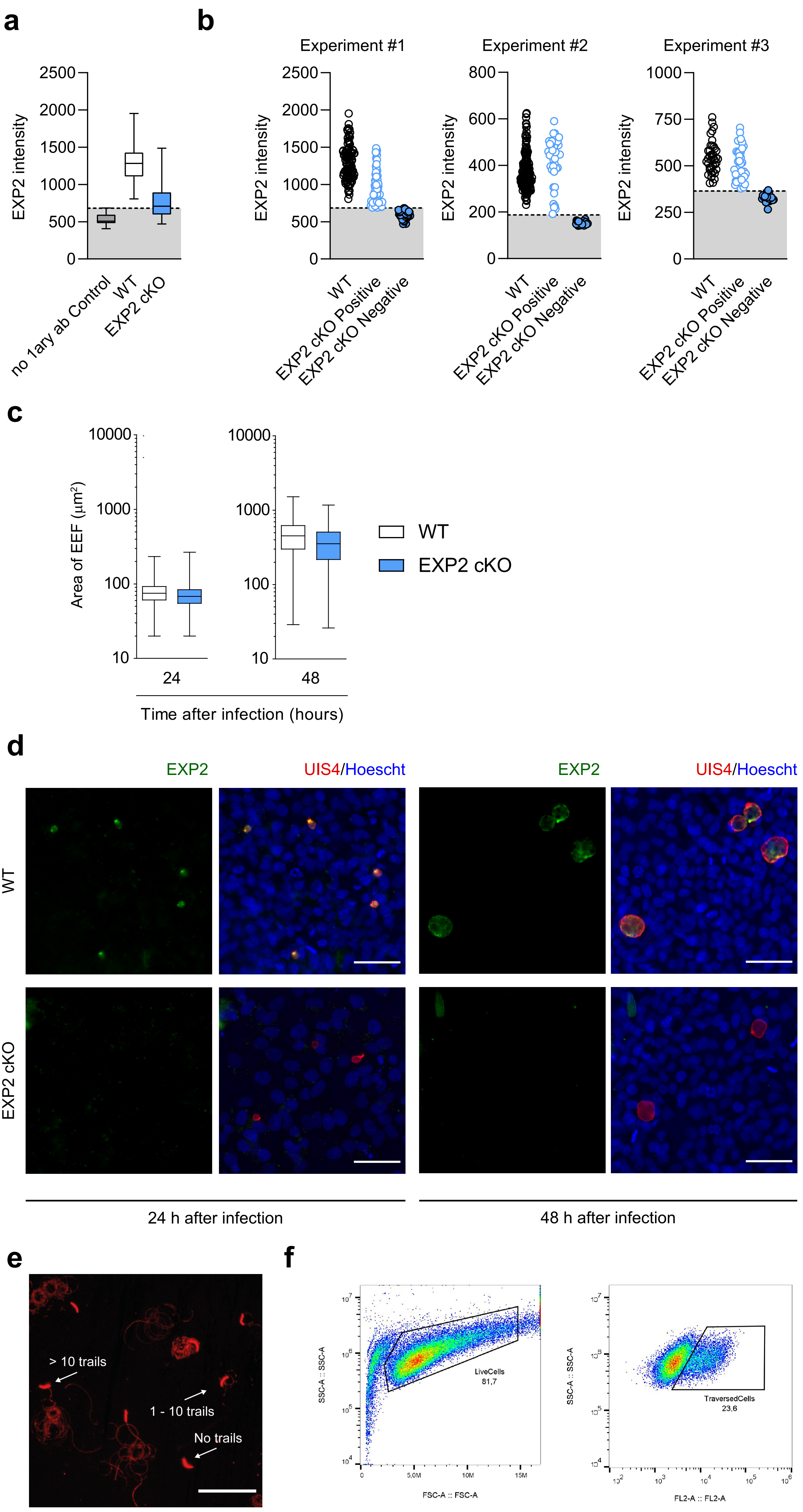

**Supplementary Fig. 1: EXP2 cKO parasites do not develop, glide and traverse differently that WT parasites.**

**a** Representative boxplot showing the range of intensities of EXP2 staining in unstained (gray), WT (white) and EXP2 cKO (blue) sporozoites. The maximum value of EXP2 intensity obtained for the unstained sporozoite sample was used as the threshold for the background of the staining (dashed line and light gray area). As such, EXP2 cKO sporozoites were then counted as being EXP2 positive (EXP2 intensity above background) and EXP2 negative (EXP2 intensity below background).

**b** Staining of EXP2 protein in WT (circles with black border) and EXP2 cKO sporozoites in 3 independent experiments, each with at least 50 sporozoites imaged. EXP2 cKO sporozoites were divided into EXP2 positive (circles with blue border) or negative (circles with black border and blue filling) based on the strategy explained on Supplementary Fig. 1a.

**c** Size of exoerythrocytic forms of WT (white) or EXP2 cKO (blue) parasites at 24 or 48h after infection (N=3 independent experiments, each with at least 200 EEFs analzed per condition).

**d** Micrographs of WT (top panel) or EXP2 cKO exoerythrocytic forms (bottom panel) at 24 (left panels) or 48 hours after infection (right panels) and stained with mouse α*Pf*EXP2 (green), α*Pb*UIS4 (red) and DNA dye Hoechst (blue). Scale bar: 50 µm. Representative images of 3 independent experiments.

**e** Representative micrograph showing WT sporozoites and the trails of CSP left in a glass coverslip after incubation for 30min at 37°C. Sporozoites and trails were stained using α*Pb*CSP protein and scored as having no trails of CSP, between 1 and 10 trails or over 10 trails, as shown in the micrograph. Scale bar: 20 µm

**f** Representative dot plots showing gating strategy used to quantify traversal WT or EXP2 cKO sporozoites through HepG2 cells using 10 kDa Rhodamine-Dextran. (N=8 independent experiments, comprising a total of 27 replicates for WT sporozoites and 26 replicates for EXP2 cKO sporozoites, in each sample, at least 10,000 cells were analyzed).

Results in **a** and **c** are shown as a boxplot (min to max distribution, where the center represents the median, the bounds of the box represent the 25th and 75th percentiles and the bounds of the whiskers represent the minimum and maximum values.).

# Supplementary Figure 2

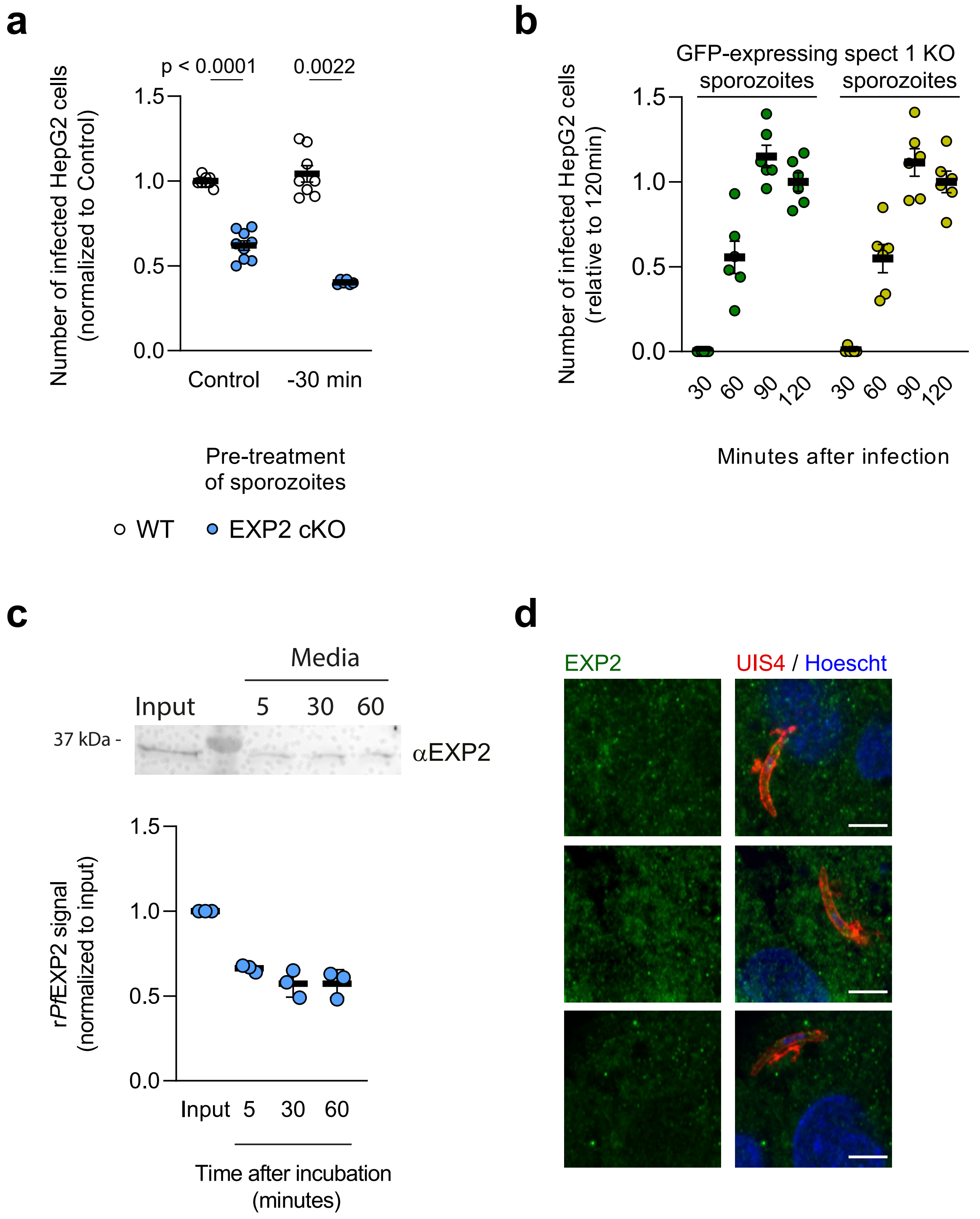

**Supplementary Fig. 2: EXP2 translocation and secretion is required only at 1 h after infection.**

**a** Number of infected cells at 2h after infection, infected with WT (white circles) or EXP2 cKO (blue circles) sporozoites, after treatment of freshly dissected sporozoite with 10 nM r*Pf*EXP2 for 30min before infection (N=3 independent experiments, comprising a total of 9 replicates).

**b** Number of infected HepG2 cells by GFP-expressing (green circles) or spect1 KO (yellow circles) sporozoites at the respective timepoint (N=3 independent experiments, comprising a total of 6 replicates, per parasite line and per time point).

**c** Western blot analysis of r*Pf*EXP2 in the presence of HepG2 cells. The supernant of HepG2 cells treated with 1 nM of rPfEXP2 for 5min, 30min and 60min. Supernatant of the cells was collected and processed for WB detection of r*Pf*EXP2, left panel. We used the same volume of sample between the different experimental conditions. r*Pf*EXP2 quantity (blue circles) was normalized to input detection, right panel. Representative WB of 3 independent experiments.

**d** Representative micrographs of invaded WT sporozoites at 2 hours after infection, stained using α*Pf*EXP2 (green), α*Pb*UIS4 (red) and the DNA dye Hoechst. Scale bar: 5 µm Representative image of 5 biological replicates, where at least 50 sporozoites were imaged per experiments.

Results in **a**, **b** and **c** are shown as mean±SEM, and two-tailed Mann-Whitney *U* test was applied for p values in **a**.

# Supplementary Figure 3

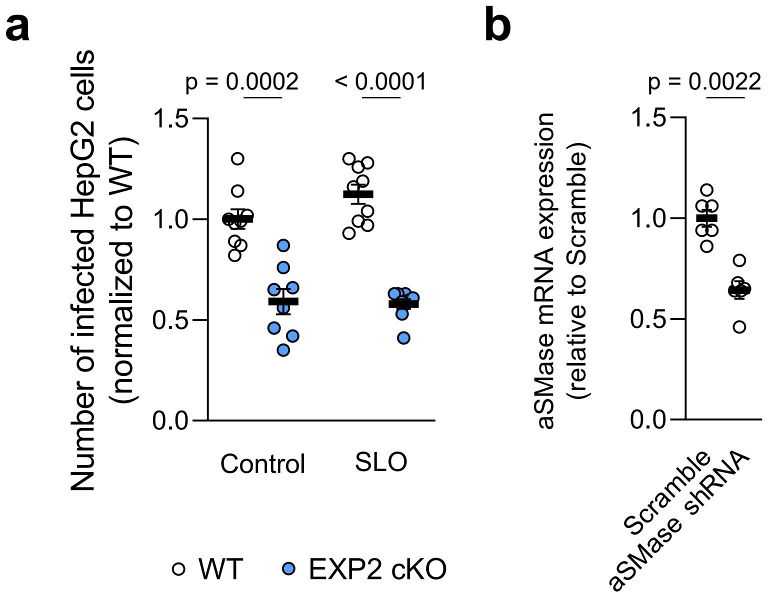

**Supplementary Fig. 3 Membrane repair pathway is required for invasion.** *Related to Fig. 3.*

**a** Number of infected cells at 2h after infection, infected with WT (white circles) or EXP2 cKO (blue circles) sporozoites, after addition of 10 nM recombinant SLO at 1h after infection (N=3 independent experiments comprising a total of 9 replicates per condition).

**b** Quantification of aSMase transcripts after transfection with either Scrambled shRNAs or shRNAs against the *SMPD1* gene (N=3 independent experiments comprising a total of 6 replicates).

Results are shown as mean±SEM, and two-tailed Mann-Whitney *U* test was applied for p value.

# Supplementary Figure 4

## Fig. 2c

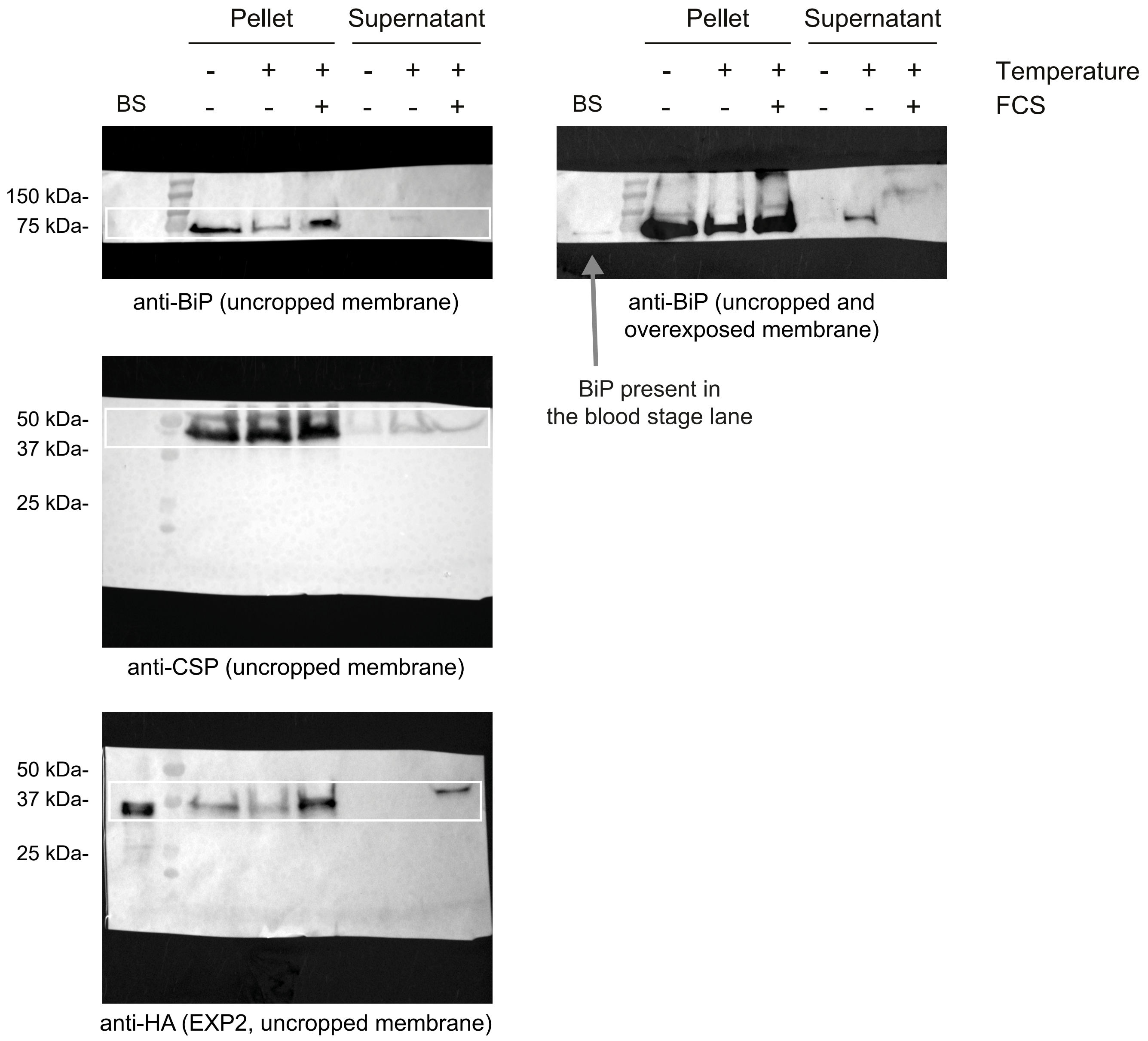


## Fig. 2c (other replicates)

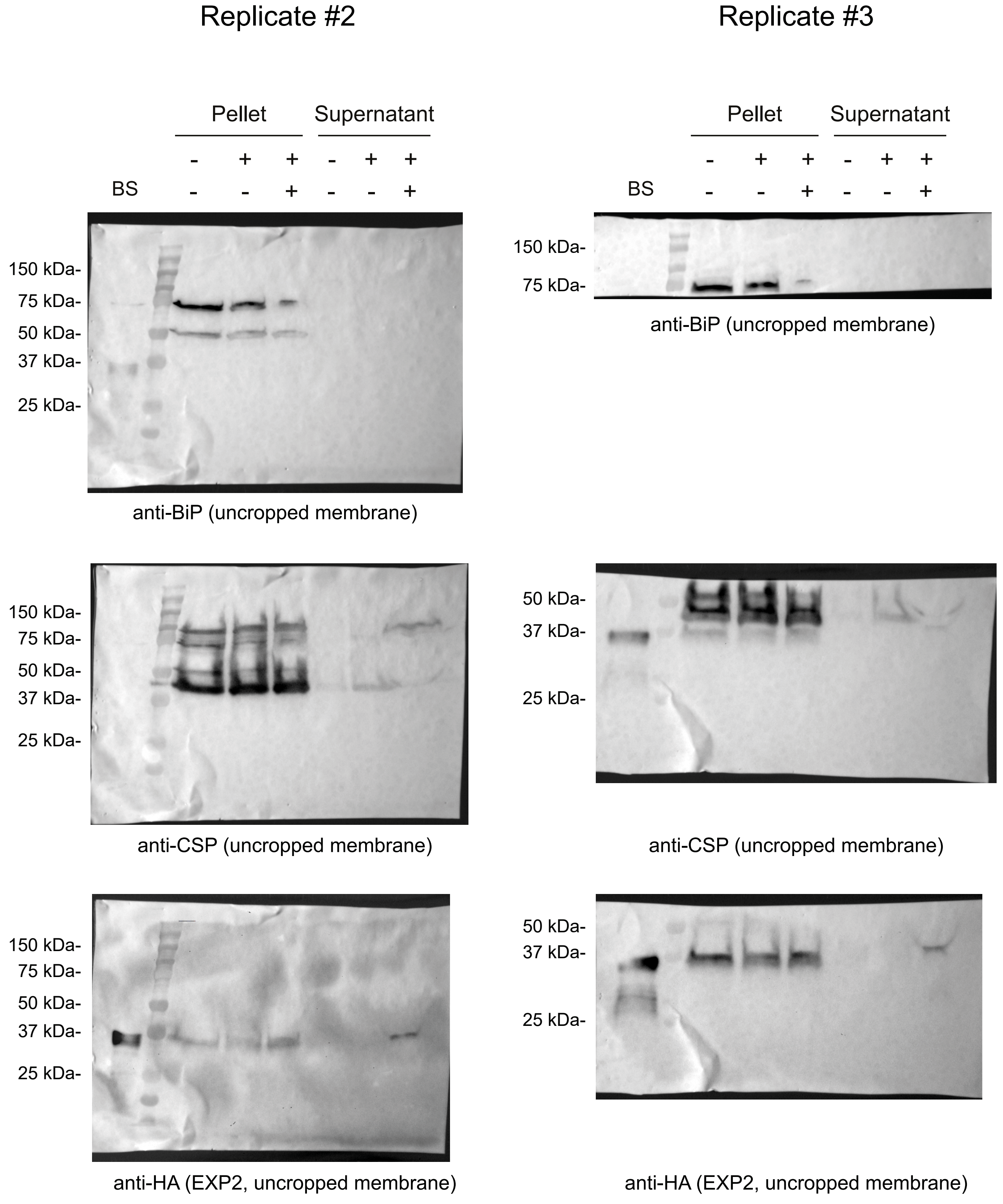

Fig. 2f

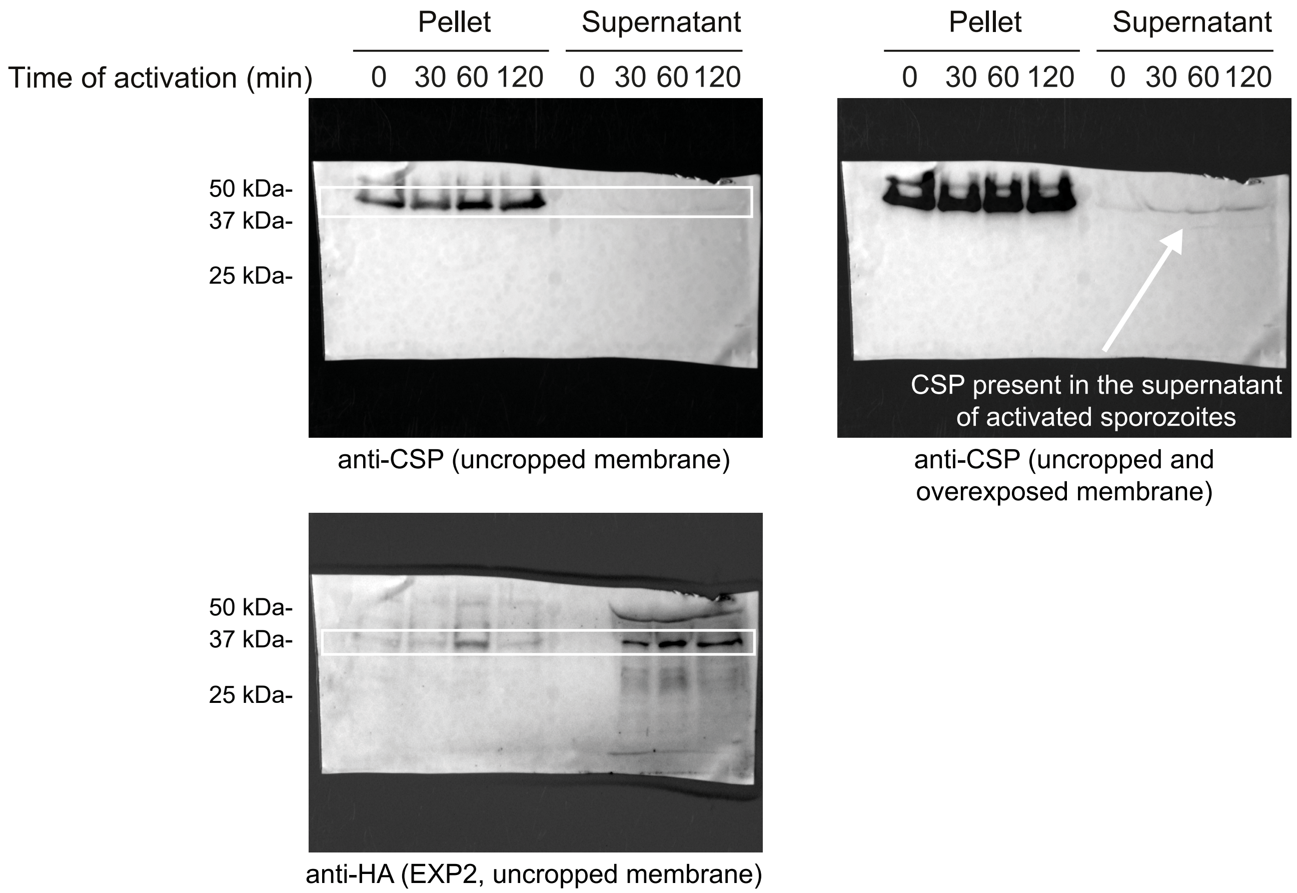


Fig. 2f (other replicate)

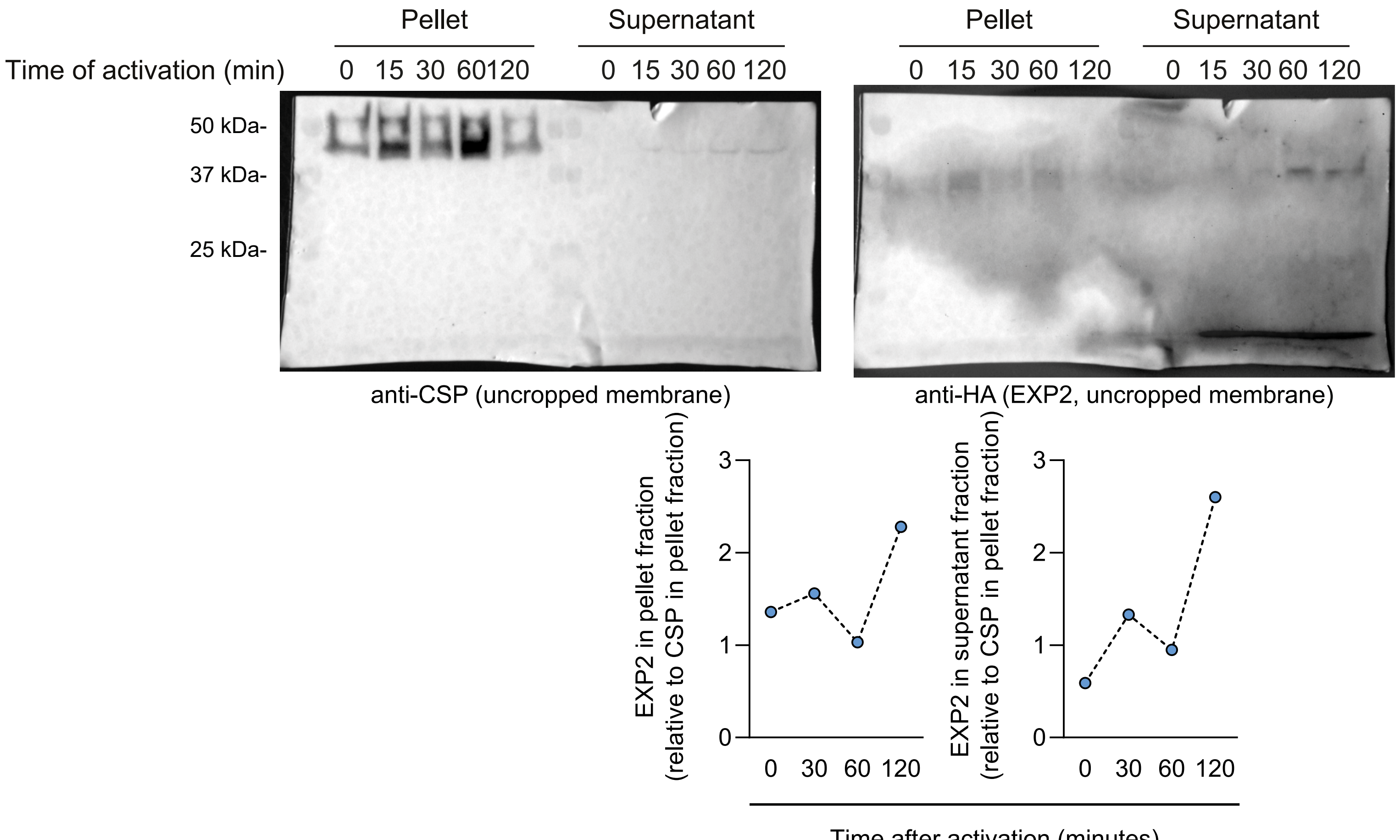

# Supplementary Fig. 2c

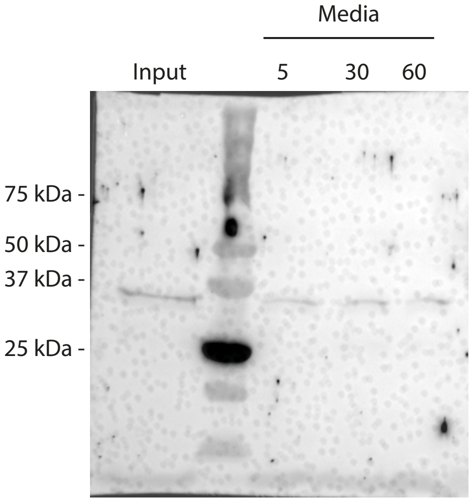


anti-EXP2 (uncropped membrane)

# Supplementary Fig. 2c (other replicates)

## Replicate #2

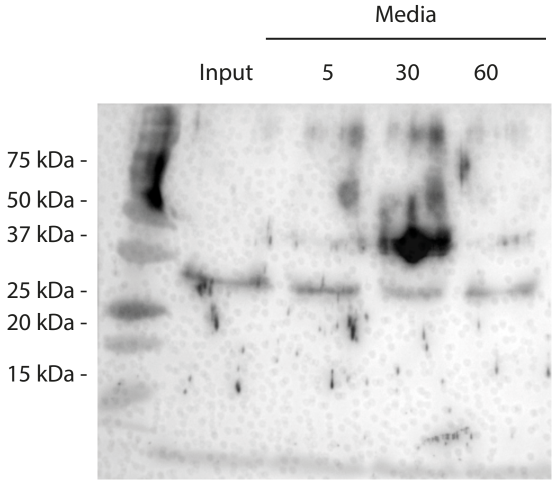


anti-EXP2 (uncropped membrane)

## Replicate #3

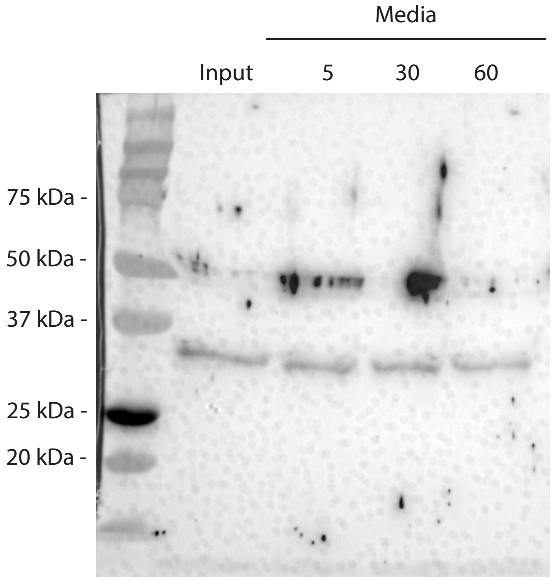


anti-EXP2 (uncropped membrane)

**Supplementary Fig. 4 – Uncropped Western Blot membranes**

Uncropped membranes shown in Fig. 2c and Fig. 2f and Supplementary Fig. 2c, with the white box showing where the images were cropped to produce the main figures. The additional replicates for these experiments are also shown.

## Supplementary Table - Complete statistical analysis

| Figure | Panel | Measurement | Comparison | Statistical Test | P value |
|---|---|---|---|---|---|
| Figure 1 | **Figure 1a** - EXP2 excision in sporozoites | qPCR of gDNA of sporozoites for detection of excised EXP2 FRT 3'UTR | WT (n=4) vs EXP2 cKO (TRAP) (n=4) | Mann-Whitney's U test | p = 0.1429 |
| | | | WT (n=4) vs EXP2 cKO (UIS4) (n=4) | Mann-Whitney's U test | p = 0.0286 |
| | **Figure 1b** - EXP2 expression in sporozoites | EXP2 intensity in sporozoites (immunofluorescence) | WT (n=7) vs EXP2 cKO (n=7) | Mann-Whitney's U test | p = 0.0159 |
| | **Figure 1c** - Liver infection | RT-qPCR of RNA of livers from infected mice for detection of *Pb* 18s rRNA | 6h WT (n=10) *vs.* EXP2 cKO (n=10) | Mann-Whitney's U test | p = 0.0056 |
| | | | 24h WT (n=15) vs. EXP2 cKO (n=15) | Mann-Whitney's U test | p = 0.0008 |
| | | | 48h WT (n=15) vs. EXP2 cKO (n=15) | Mann-Whitney's U test | p = 0.0037 |
| | **Figure 1d** - HepG2 cells infection | Number of infected HepG2 cells (immunofluorescence) | 2h WT (n=12) vs. EXP2 cKO (n=12) | Mann-Whitney's U test | p = 0.0001 |
| | | | 24h WT (n=15) vs. EXP2 cKO (n=15) | Mann-Whitney's U test | p = 0.0043 |
| | | | 48h WT (n=15) vs. EXP2 cKO (n=15) | Mann-Whitney's U test | p = 0.0024 |
| | **Figure 1e** - Correlation between EXP2 excision and invasion | Comparison between qPCR of gDNA of sporozoites for detection of excised EXP2 FRT 3'UTR *versus* Number of infected HepG2 cells (immunofluorescence) for each individual experiment | EXP2 excision vs Invasion rate (n=10) | Pearson's R | R = 0.8800 |
| | **Figure 1f** - EXP2 expression in exoerythrocytic forms | EXP2 intensity in EEFs (immunofluorescence) | 24h WT (n=3) vs EXP2 cKO (n=3) | Mann-Whitney's U test | p = 0.0159 |
| | | | 48h WT (n=3) vs EXP2 cKO (n=3) | Mann-Whitney's U test | p = 0.0022 |
| | **Figue 1g** - Gliding motility | Number of CSP trails in coverslip (immunofluorescence) | WT (n=3) vs EXP2 cKO (n=3) | $\chi^2$ test | p = 0.5407 |
| | **Figue 1h** - Traversal ability | Number of dextran-positive HepG2 cells (flow cytometry) | WT (n=27) vs EXP2 cKO (n=26) | Mann-Whitney's U test | p = 0.9262 |
| | **Figure 1i** - In and out | Number of all CSP-positive sporozoites (immunofluorescence) | Intracellular WT (n=6) vs EXP2 cKO (n=7) | Mann-Whitney's U test | p > 0.9999 |
| | | Number of UIS4-positive (intracellular)sporozoites (immunofluorescence) | Intracellular WT (n=6) vs EXP2 cKO (n=7) | Mann-Whitney's U test | p = 0.0022 |
| | | Number of only CSP-positive (extracellular) sporozoites (immunofluorescence) | Extracellular WT (n=6) vs EXP2 cKO (n=7) | Mann-Whitney's U test | p = 0.0206 |
| Figure 2 | **Figure 2 d** - rEXP2 rescue | Number of infected HepG2 cells (immunofluorescence) | Control WT (n=9) vs EXP2 cKO (n=9) | Mann-Whitney's U test | p < 0.0001 |
| | | | Cells treated at 0 h WT (n=9) vs EXP2 cKO (n=9) | Mann-Whitney's U test | p < 0.0001 |
| | | | Cells treated at 1 h WT (n=9) vs EXP2 cKO (n=9) | Mann-Whitney's U test | p = 0.6907 |
| | **Figure 2 e** - Temporal dynamics of mRNA of invasion-related genes (EXP2) | RT-qPCR of RNA of HepG2 cells and sporozoites for detection of *EXP2* rRNA | 30min (n=6) vs 60min (n=6) | Mann-Whitney's U test | p = 0.0087 |
| | | | 30min (n=6) vs 120min (n=6) | Mann-Whitney's U test | p = 0.6688 |
| | | | 60min (n=6) vs 120min (n=6) | Mann-Whitney's U test | p = 0.0130 |
| | **Figure 2 e** - Temporal dynamics of mRNA of invasion-related genes (GAP45) | RT-qPCR of RNA of HepG2 cells and sporozoites for detection of *GAP45* rRNA | 30min (n=6) vs 60min (n=6) | Mann-Whitney's U test | p = 0.4177 |
| | | | 30min (n=6) vs 120min (n=6) | Mann-Whitney's U test | p = 0.1320 |
| | | | 60min (n=6) vs 120min (n=6) | Mann-Whitney's U test | p = 0.1926 |
| | **Figure 2 e** - Temporal dynamics of mRNA of invasion-related genes (EXP1) | RT-qPCR of RNA of HepG2 cells and sporozoites for detection of *EXP1* rRNA | 30min (n=6) vs 60min (n=6) | Mann-Whitney's U test | p = 0.4848 |
| | | | 30min (n=6) vs 120min (n=6) | Mann-Whitney's U test | p = 0.0931 |
| | | | 60min (n=6) vs 120min (n=6) | Mann-Whitney's U test | p = 0.3095 |
| Supplementary Figure 2 | **Supplementary Figure 2a** - rEXP2 pre-treatment of sporozoites | Number of infected HepG2 cells (immunofluorescence) | Control WT (n=9) vs EXP2 cKO (n=9) | Mann-Whitney's U test | p < 0.0001 |
| | | | Pre-treated sporozoites WT (n=9) vs EXP2 cKO (n=9) | Mann-Whitney's U test | p = 0.0022 |
| Figure 3 | **Figure 3 a** - rEXP2 rescue | Number of infected HepG2 cells (immunofluorescence) | Control WT (n=15) vs EXP2 cKO (n=15) | Mann-Whitney's U test | p < 0.0001 |
| | | | 0.5 nM rEXP2 WT (n=9) vs EXP2 cKO (n=9) | Mann-Whitney's U test | p = 0.0294 |
| | | | 1 nM rEXP2 WT (n=15) vs EXP2 cKO (n=15) | Mann-Whitney's U test | p = 0.0303 |
| | | | 10 nM rEXP2 WT (n=15) vs EXP2 cKO (n=15) | Mann-Whitney's U test | p = 0.6907 |
| | **Figure 3 b** - r$\alpha_{HL}$ rescue | Number of infected HepG2 cells (immunofluorescence) | Control WT (n=9) vs EXP2 cKO (n=9) | Mann-Whitney's U test | p < 0.0001 |
| | | | 0.5 nM rEXP2 WT (n=9) vs EXP2 cKO (n=9) | Mann-Whitney's U test | p = 0.0106 |
| | | | 1 nM rEXP2 WT (n=9) vs EXP2 cKO (n=9) | Mann-Whitney's U test | p = 0.6835 |
| | | | 10 nM rEXP2 WT (n=9) vs EXP2 cKO (n=9) | Mann-Whitney's U test | p > 0.9999 |
| | **Figure 3 e** - Desipramine titration (n=9) | Number of infected HepG2 cells (immunofluorescence) | n/a | Top value | 1.0770 |
| | | | | Bottom value | 0.0009 |
| | | | | $IC_{50}$ value | 14.95 |
| | | | | Slope | -0.0691 |
| | **Figure 3 f** - aSMase knock-down | Number of infected HepG2 cells (immunofluorescence) | Control Scramle (n=9) vs aSMase shRNA (n=9) | Mann-Whitney's U test | p = 0.0022 |
| | | | aSMase treated Scramle (n=9) vs aSMase shRNA (n=9) | Mann-Whitney's U test | p = 0.1667 |
| | | | aSMase shRNA Control (n=9) vs *Bc.* aSMase (n=9) | Mann-Whitney's U test | p = 0.0022 |
| | **Figure 3 g** - aSMase titration | Number of infected HepG2 cells (immunofluorescence) | Control WT (n=15) vs EXP2 cKO (n=15) | Mann-Whitney's U test | p < 0.0001 |
| | | | aSMase WT (n=15) vs EXP2 cKO (n=15) | Mann-Whitney's U test | p = 0.1339 |
| Supplementary Figure 3 | **Supplementary Figure 3 a** - SLO rescue experiments | Number of infected HepG2 cells (immunofluorescence) | Control WT (n=9) vs Control EXP2 cKO (n=9) | Mann-Whitney's U test | p = 0.0001 |
| | | | Control EXP2 cKO (n=9) vs SLO-treated EXP2 cKO (n=9) | Mann-Whitney's U test | p = 0.2856 |
| | **Supplementary Figure 3 b** - shRNA aSMase knock-down efficiency | RT-qPCR of RNA of HepG2 cells transfected with Scramble or SMPD1 shRNA for detection of *HsSMPD1* RNA | Control WT (n=6) vs EXP2 cKO (n=6) | Mann-Whitney's U test | p = 0.0022 |
