# Supplementary material for: Plasmodium translocon component EXP2 facilitates hepatocyte invasion
Source: Nat Commun. 2020 Nov 6;11:5654. doi: 10.1038/s41467-020-19492-4 (PMC7648069; doi:10.1038/s41467-020-19492-4)
Supplement: Supplementary file 3 — Reporting Summary [file 41467_2020_19492_MOESM3_ESM.pdf]

nature research

Corresponding author(s): Maria M Mota & Vanessa Zuzarte-Luís

Last updated by author(s): Sep 30, 2020

Reporting Summary

Nature Research wishes to improve the reproducibility of the work that we publish. This form provides structure for consistency and transparency in reporting. For further information on Nature Research policies, see Authors & Referees and the Editorial Policy Checklist .

Statistics

For all statistical analyses, confirm that the following items are present in the figure legend, table legend, main text, or Methods section.

n/a	Confirmed	
☐	☒	The exact sample size (*n*) for each experimental group/condition, given as a discrete number and unit of measurement
☐	☒	A statement on whether measurements were taken from distinct samples or whether the same sample was measured repeatedly
☐	☒	The statistical test(s) used AND whether they are one- or two-sided<br>*Only common tests should be described solely by name; describe more complex techniques in the Methods section.*
☒	☐	A description of all covariates tested
☒	☐	A description of any assumptions or corrections, such as tests of normality and adjustment for multiple comparisons
☐	☒	A full description of the statistical parameters including central tendency (e.g. means) or other basic estimates (e.g. regression coefficient) AND variation (e.g. standard deviation) or associated estimates of uncertainty (e.g. confidence intervals)
☐	☒	For null hypothesis testing, the test statistic (e.g. *F*, *t*, *r*) with confidence intervals, effect sizes, degrees of freedom and *P* value noted<br>*Give P values as exact values whenever suitable.*
☒	☐	For Bayesian analysis, information on the choice of priors and Markov chain Monte Carlo settings
☒	☐	For hierarchical and complex designs, identification of the appropriate level for tests and full reporting of outcomes
☐	☒	Estimates of effect sizes (e.g. Cohen's *d*, Pearson's *r*), indicating how they were calculated

Our web collection on statistics for biologists contains articles on many of the points above.

Software and code

Policy information about availability of computer code

Data collection	To acquire microscopy data, Metamorph (v7.7.9.0) and ZEN 2 (Blue version), Leica DFC Twain (v7.7.1) were used.<br>To quantify mRNA abundance, 7500Fast (v2.3) and QuantStudio (v1.3) were used.<br>To acquire Flow Cytometry data, Accuri C6 software (v1.0.264.21) were used.<br>To acquire WB images, ImageLab (v5.2.1) were used.
Data analysis	For data analysis, GraphPad Prism (version 5) were used.<br>For image analysis, FIJI (version 1.52i).<br>For flow cytometry analysis, FlowJo X was used.<br>For data presentation, Adobe Illustrator (version CS4) was used.

For manuscripts utilizing custom algorithms or software that are central to the research but not yet described in published literature, software must be made available to editors/reviewers. We strongly encourage code deposition in a community repository (e.g. GitHub). See the Nature Research guidelines for submitting code & software for further information.

Data

Policy information about availability of data

All manuscripts must include a data availability statement. This statement should provide the following information, where applicable:
- Accession codes, unique identifiers, or web links for publicly available datasets
- A list of figures that have associated raw data
- A description of any restrictions on data availability

Source data used for producing the figures in this manuscript and in the Supplementary Information are provided with this paper.
Replicates of Western Blots are provided in Supplementary Figure 4. All other data are available from the authors upon reasonable request.
Source data are provided with this paper.

Field-specific reporting

Please select the one below that is the best fit for your research. If you are not sure, read the appropriate sections before making your selection.

- [x] Life sciences
- [ ] Behavioural & social sciences
- [ ] Ecological, evolutionary & environmental sciences

For a reference copy of the document with all sections, see nature.com/documents/nr-reporting-summary-flat.pdf

Life sciences study design

All studies must disclose on these points even when the disclosure is negative.

Sample size	No statistical methods were used to determine sample size. Sample size is always specified in every figure legend and experiments were generally repeated at least three times. Samples sizes are consistent with similar published studies.
Data exclusions	No data points were excluded from analysis.
Replication	All attempts of replication were successfull, the observed variation is reported and contributes to the statistical calculation. Experiments were generally repeated at least three times, independently.
Randomization	Experimental animals were randomly assign to the different experimental groups. Assignement of treatment groups and strains was randomized between biological replicates. Microscopy analysis was performed in randomly acquired images.
Blinding	Investigators were not blinded to the group allocation. However, data collection was performed in an unbiased manner and the analysis were performed on quantitative endpoints that are not subject to investigator bias.

Reporting for specific materials, systems and methods

We require information from authors about some types of materials, experimental systems and methods used in many studies. Here, indicate whether each material, system or method listed is relevant to your study. If you are not sure if a list item applies to your research, read the appropriate section before selecting a response.

Materials & experimental systems

n/a	Involved in the study	
☐	☒	Antibodies
☐	☒	Eukaryotic cell lines
☒	☐	Palaeontology
☐	☒	Animals and other organisms
☒	☐	Human research participants
☒	☐	Clinical data

Methods

n/a	Involved in the study	
☒	☐	ChIP-seq
☐	☒	Flow cytometry
☒	☐	MRI-based neuroimaging

Antibodies

Antibodies used	All antibodies used in this study are detailed in the Methods sections, in the "Immunofluorescence of hepatoma cells", "Sporozoite staining", "Gliding Assays", "Western Blot" and "Electron Microscopy" subsections.<br>- Mouse antiCSP (clone 3D11, MR4)<br>- Mouse antiEXP2 (clone 7.7, European Malaria Reagent Repository)<br>- Rabbit antiEXP2 (a gift from the lab of Paul Gilson, custom antibody)<br>- Goat antiUIS4 (AB0042-500, Sicgen)<br>- Rabbit antiTRAP (a gift from the lab of Joana Tavares, custom antibody)<br>- Mouse antiRON4 (a gift from the lab of Maryse Lebrun, custom antibody
Validation	All antibodies have been previously validated:<br>- Mouse antiCSP (Yoshida, et al., Science, 1980)<br>- Mouse antiEXP2 (Hall, et al., Mol Biochem Parasitol, 1983)<br>- Rabbit antiEXP2 (Bullen, et al., J Biol Chem, 2012)<br>- Goat antiUIS4 (Itoe, et al., Cell Host Microbe, 2014)<br>- Rabbit antiTRAP (Matuschewski, et al., EMBO Journal, 2002)<br>- Mouse antiRON4 (Suarez, et al., Nat Commun, 2019

Eukaryotic cell lines

Policy information about cell lines

Cell line source(s)	HepG2 cells used in this study were obtained from ATCC.
Authentication	The cell line was not authenticated.
Mycoplasma contamination	Cells used were negative for Mycoplasm contamination.
Commonly misidentified lines (See ICLAC register)	No commonly misidentified cell lines were used in this project.

Animals and other organisms

Policy information about studies involving animals; ARRIVE guidelines recommended for reporting animal research

Laboratory animals	For this manuscript, C57Bl/6J and BALB/c mice were used, purchased from Charles River Laboratories.<br>All mice used were males, with 6-8 weeks of age
Wild animals	No wild animals were used in this study.
Field-collected samples	No filed-collected samples were used in this study.
Ethics oversight	All in vivo protocols were approved by the ORBEA committee of the iMM JLA and were performed according to national and European regulations.

Note that full information on the approval of the study protocol must also be provided in the manuscript.

Flow Cytometry

Plots

Confirm that:

- [x] The axis labels state the marker and fluorochrome used (e.g. CD4-FITC).
- [x] The axis scales are clearly visible. Include numbers along axes only for bottom left plot of group (a 'group' is an analysis of identical markers).
- [x] All plots are contour plots with outliers or pseudocolor plots.
- [x] A numerical value for number of cells or percentage (with statistics) is provided.

Methodology

Sample preparation	To quantify the level of traversal, HepG2 cells and sporozoites were incubated in the presence of 0.5 mg/mL of 10 kDa Dextran-Rhodamine (Thermo Fisher Scientific) in complete DMEM at 37°C. The dextran molecule is passively taken up by cells that have been traversed and is detected because of the Rhodamine dye, that has Excitation/Emission maxima at 570/590nm. Data collection was performed using BD Accuri C6 cytometer (Franklin Lakes, New Jersey, USA) and Accuri C6 software (v1.0.264.21) software was used. Data analysis was performed using FlowJo X software (FlowJo LLC, Ashland, OR, USA) (see Supplementary Fig. 1f for the gating strategy used).
Instrument	A BD Accuri C6 cytometer was used.
Software	FlowJo version X was used.
Cell population abundance	Traversed cells would correspond from 10 to 20% of the ungated sample.
Gating strategy	Live cells were gated on FSC-A/SSC-A (SSC-A on log scale). Next, traversed cells were gated using FL2-A/SSC-A (SSC-A on log scale), using the FL2-A-positive (right side of the plot).<br>Cells that were cultured without dextran-rhodamine were used as unstained sample, to adjust the gating of traversed cells.<br>Gating strategy is explained in Supplementary Figure 1f

- [x] Tick this box to confirm that a figure exemplifying the gating strategy is provided in the Supplementary Information.
